# Functional Characterization of *CLCN4* Variants Associated With X-Linked Intellectual Disability and Epilepsy

**DOI:** 10.3389/fnmol.2022.872407

**Published:** 2022-05-31

**Authors:** Raul E. Guzman, Juan Sierra-Marquez, Stefanie Bungert-Plümke, Arne Franzen, Christoph Fahlke

**Affiliations:** Institute of Biological Information Processing, Molecular and Cellular Physiology (IBI-1), Forschungszentrum Jülich, Jülich, Germany

**Keywords:** ion channels and epilepsy, *CLCN4*, patch clamp, chloride-proton exchanger, intellectual disability

## Abstract

Early/late endosomes, recycling endosomes, and lysosomes together form the endo-lysosomal recycling pathway. This system plays a crucial role in cell differentiation and survival, and dysregulation of the endo-lysosomal system appears to be important in the pathogenesis of neurodevelopmental and neurodegenerative diseases. Each endo-lysosomal compartment fulfils a specific function, which is supported by ion transporters and channels that modify ion concentrations and electrical gradients across endo-lysosomal membranes. CLC-type Cl^–^/H^+^ exchangers are a group of endo-lysosomal transporters that are assumed to regulate luminal acidification and chloride concentration in multiple endosomal compartments. Heterodimers of ClC-3 and ClC-4 localize to various internal membranes, from the endoplasmic reticulum and Golgi to recycling endosomes and late endosomes/lysosomes. The importance of ClC-4-mediated ion transport is illustrated by the association of naturally occurring *CLCN4* mutations with epileptic encephalopathy, intellectual disability, and behavioral disorders in human patients. However, how these mutations affect the expression, subcellular localization, and function of ClC-4 is insufficiently understood. We here studied 12 *CLCN4* variants that were identified in patients with X-linked intellectual disability and epilepsy and were already characterized to some extent in earlier work. We analyzed the consequences of these mutations on ClC-4 ion transport, subcellular trafficking, and heterodimerization with ClC-3 using heterologous expression in mammalian cells, biochemistry, confocal imaging, and whole-cell patch-clamp recordings. The mutations led to a variety of changes in ClC-4 function, ranging from gain/loss of function and impaired heterodimerization with ClC-3 to subtle impairments in transport functions. Our results suggest that even slight functional changes to the endosomal Cl^–^/H^+^ exchangers can cause serious neurological symptoms.

## Introduction

The endo-lysosomal system degrades and recycles internalized material and damaged cell components and is involved in repairing the plasma membrane and releasing non-degradable material (Medina et al., 2011). Pronounced differences in luminal pH suggest that luminal acidification is a major determinant of the functional specificity of endo-lysosomal compartments. Acidification of endosomes and lysosomes is driven by the H^+^-ATPase and regulated by a variety of ion channels and transporters. Five CLC transporters are expressed in endosomal membranes, with isoform-specific localization and functions (Stauber and Jentsch, 2010; Bose et al., 2021). The transporters utilize the stoichiometrically coupled exchange of two Cl^–^ ions with one H^+^ ion to regulate and maintain endosomal pH and Cl^–^ concentration (Ishida et al., 2013; Stauber and Jentsch, 2013). ClC-3 and ClC-4 are found in various human organs, with strong expression in the brain. Alternative splicing of *CLCN3* results in the targeting of ClC-3 variants to the Golgi, recycling and late endosomes, and lysosomes (Guzman et al., 2015). ClC-4 homodimers are mainly found in the endoplasmic reticulum (ER), whereas heterodimerization with ClC-3 targets ClC-4 subunits to recycling and late endosomes/lysosomes (Guzman et al., 2017). ClC-5 is expressed in early endosomes in the kidney, where it regulates endocytic uptake in the proximal tubule (Jentsch and Pusch, 2018; Sakhi et al., 2021). ClC-6 and ClC-7 localize to late endosomes and lysosomes (Jentsch and Pusch, 2018).

Animal models lacking ClC-4 do not exhibit an apparent phenotype (van Slegtenhorst et al., 1994; Rickheit et al., 2010; Weinert et al., 2020), in contrast to the severe phenotypes caused by genetic ablation of the other CLC exchangers (Piwon et al., 2000; Kornak et al., 2001; Stobrawa et al., 2001; Dickerson et al., 2002; Yoshikawa et al., 2002; Poet et al., 2006). Sequence variations in *CLCN4* have recently been associated with X-linked intellectual disability, epilepsy, white matter abnormality, and cortical atrophy in humans (Veeramah et al., 2013; Hu et al., 2016; Palmer et al., 2018; Zhou et al., 2018; He et al., 2021; Xu et al., 2021). So far, more than 20 different *CLCN4* mutations have been identified. Several disease-associated *CLCN4* mutations were tested by electrophysiological analysis after heterologous expression in oocytes (Hu et al., 2016; Palmer et al., 2018). These experiments revealed reduced macroscopic current amplitudes, however, no attempts were done to distinguish altered trafficking from impaired transport function or to identify mechanisms underlying reduced anion transport. Although endosomal targeting of ClC-4 requires association to ClC-3 (Guzman et al., 2017; Weinert et al., 2020), only homodimers were studied and potential changes in hetero-oligomerization were ignored. Here, we studied the functional consequences of 12 disease-associated *CLCN4* mutations in a mammalian heterologous expression system using whole-cell patch clamping, confocal imaging, and denaturing and native gel electrophoresis. We report changes in transport, subcellular localization, protein stability, and oligomerization capacity of the ClC-4 variants.

## Materials and Methods

### Plasmid Construction

cDNAs encoding full-length WT human ClC-4 (Alekov and Fahlke, 2009) or mouse ClC-3b (Guzman et al., 2015) were cloned into FsY1.1 G.W. or p156rrL vectors (kindly provided by Dr. Mikhail. Filippov, Nizhny Novgorod, Russia, and Dr. Dieter. Bruns, Homburg, Germany). Enhanced green or monomeric cherry fluorescent proteins (eGFP or mCherry) were fused in frame to the 5′ end of the coding sequence of each CLC transporter. Overlapping PCR strategies were used to introduce the ClC-4 mutations and to generate chimeric constructs. All constructs were verified by sequencing the complete open reading frame, and two independent recombinants from each transformation were tested for possible functional differences. To help distinguish ClC-3b and ClC-4 electrophoretically, we increased the molecular weight of ClC-3b by adding the coding sequence of maltose-binding protein (MBP) in frame to its 3′ end. We also inserted point mutations to substitute glutamine at N880 and N883 to prevent MBP -ClC-3b glycosylation.

### Electrophysiological Experiments

For electrophysiological recordings, HEK293T cells were transfected with plasmids encoding WT or mutant ClC-4-eGFP fusion proteins using the calcium phosphate method (Garcia-Olivares et al., 2008). Only fluorescent cells were studied by whole-cell patch-clamp recordings with an EPC-10 amplifier controlled by PatchMaster (HEKA Elektronik, Harvard Bioscience, Reutlingen, Germany) (Guzman et al., 2013). Images were taken with an Andor’s Neo 5.5 sCMOS camera and analyzed using ImageJ 1.44p software (Image J v.1.53c, Wayne Rasband, National Institutes of Health, Bethesda, Rockville, MD, United States) (Schneider et al., 2012). Borosilicate pipettes (GC150F-10, Harvard Apparatus, Holliston, MA, United States) were pulled with resistances of 1.0–2.0 MΩ; in all experiments, capacitance cancelation and 80–85% series resistance compensation were applied to ensure a voltage error of below 5 mV. Currents were elicited by applying 10 ms test pulses (–115 mV to +175 mV in 10 mV increment every 500 ms) from a holding potential of 0 mV and digitized with 100 kHz sampling rates. For all representative recordings, P/8 leak subtraction with a baseline potential of -30 mV was used to cancel linear capacitances (Bezanilla and Armstrong, 1977). We carefully tested all mutant proteins for potential alterations in the current response to negative voltages. For electrophysiological experiments, bath solutions contained (in mM) 145 NaCl, 15 HEPES, 4 K-gluconate, 2 CaCl_2_, and 1 MgCl_2_, pH 7.4, and internal recording solutions contained (in mM) 120 NaCl, 15 HEPES, 5 MgCl_2_, 5 EGTA, and 5 Na-ATP, pH 7.4.

### Confocal Microscopy and Image Analysis

For confocal imaging, HEK293T cells were co-transfected with the WT or mutant ClC-4-eGFP fusion construct and a plasmid encoding fluorescent calnexin or ClC-3b-mCherry (Guzman et al., 2015. We received the ER marker calnexin as gift from Michael Davidson (Addgene plasmid # 55005^1^; RRID:Addgene_55005). Cells were plated on poly-L-lysine-coated coverslips at 24 h after transfection, and images were taken 24 h later with a Leica TCS SP5 II inverted microscope (Leica Microsystems, Wetzlar, Germany) using a 63 × /1.40 NA oil immersion objective in phosphate-buffered saline at room temperature. Images were digitalized with a resolution of 1024 × 1024 pixels, 200 Hz velocity, and 6-line average in sequential scanning mode. eGPF was excited with a 488-nm Ar-laser and mCherry with a 594-nm He-Ne laser. Emission signals were detected after filtering with a 500–550 or 600–650 nm bandpass filter. Confocal images were processed for publications using ImageJ 1.44p software (Image J v.1.53c, Wayne Rasband, National Institutes of Health, Bethesda, Rockville, MD, United States) (Schneider et al., 2012).

### Biochemical Analysis

HEK293T cells were transfected with plasmids encoding WT or mutant ClC-4-eGFP fusion proteins with or without a plasmid encoding the glycosylation-defective mutant, MBP-ClC-3b-eGFP N880/883Q. Transfected HEK293T cells were washed with ice-cold phosphate-buffered saline and lysed with buffer containing 0.1 M sodium phosphate, pH 8.0, 0.5% digitonin, protease inhibitors, and 20 mM iodoacetamide, as previously described (Guzman et al., 2017). Approximately 10 μg whole-cell lysate was analyzed by reducing 10% SDS-PAGE (Laemmli, 1970) at room temperature for approximately 2 h at 18 mA. For high-resolution clear native gel electrophoresis (hrCNE), native 4–14% acrylamide gradient gels were prepared as previously described (Nicke et al., 1998; Wittig et al., 2007); the anode buffer contained 25 mM imidazole/HCl, pH 7.0, and the cathode buffer contained 50 mM tricine, 7.5 mM imidazole, pH 7.0, the anionic detergent DOC (0.05%), and the non-ionic detergent DDM (0.01%) (Wittig et al., 2007). Approximately 10 μg whole-cell lysate samples were run at 8°C for a total of 3 h (100 V for 1 h, followed by 150 V for 2 h).

Protein bands were visualized using a fluorescence gel scanner (Typhoon FLA9500, GE Healthcare, Freiburg, Germany) at 100 μm resolution. eGFP was excited at 473 nm and emissions were recorded using a 530/20 bandpass filter. Gel images were quantified using ImageJ 1.44p software (Schneider et al., 2012). Gels were analyzed in black and white and the appearance of the entire gel was adjusted using the Brightness and Contrast tool of ImageJ. For glycosylation analysis, a rectangular ROI (region of interest) was selected between apparent molecular weights of 80–140 kDa to cover bands containing non-glycosylated or glycosylated WT ClC-4-eGFP or mutant protein in the absence or presence of MBP-ClC-3b-eGFP N880/883Q. Within these ROIs, the intensity of glycosylated and non-glycosylated ClC-4-eGFP bands was determined and used to calculate the percentage of glycosylated ClC-4-eGFP molecules. The total protein was calculated as the sum of intensities of the glycosylated and non-glycosylated ClC-4-eGFP bands. To quantify the oligomerization capacity of ClC-4 mutant variants, a rectangular ROI was selected that covered all ClC-3b or ClC-4 homodimeric or heterodimeric assemblies of WT/mutant protein bands (Supplementary Figure 1). Total protein was calculated as the sum of intensities of all expressed proteins, and intensities of ClC-3b/ClC-4 WT/mutant heterodimeric bands divided by total fluorescence were used to quantify the percentage of heterodimers. Bands from experiments with transfections of ClC-3b or ClC-4 alone were used to appropriately assign protein bands in co-expression experiments.

For PNGaseF or EndoH treatment, 10 μg whole-cell lysate was incubated with 0.5 μl enzyme at 30°C for 30 min. The glycosylation states of ClC proteins were analyzed by reducing 10% SDS-PAGE and subsequent Typhoon scanning.

### Homology Modeling Structure of ClC-4

Atomic-resolution structures of a human ClC-4 dimer (UniProt ID P51793) were generated using the neural network-based model AlphaFold-Multimer (Evans et al., 2021), a recent improvement of AlphaFold2 (Jumper et al., 2021) trained to predict multimeric protein structures. We generated 20 ClC-4 models and selected the best-ranked model according to model confidence as described (Evans et al., 2021). A local installation of AlphaFold-Multimer (version 2.1.1^2^) was used on Linux servers equipped with four Nvidia A100 GPUs.

### Data Analysis

Data were analyzed using a combination of FitMaster (HEKA), Origin (OriginLab, Northampton, United States), SigmaPlot (Systat Software, Düsseldorf, Germany), and Excel (Microsoft, Redmond, WAS, United States) software. All summary data are given as mean ± s.e.m (standard error of the mean). Data are graphically presented as mean ± standard error of the mean (s.e.m) or box-whisker plots indicating the upper and the lower quartiles and whiskers the upper and lower 90%. The comparison was made using ANOVA after passing assumptions of normality (Shapiro–Wilk test) and equal variances (Levene’s test) or Mann–Whitney Rank Sum test with *p < 0.05, ^**^p < 0.01, ^***^p < 0.001 levels of significance.

## Results

### Most Disease-Causing Mutations Do Not Affect Protein Expression or Stability

**FIGURE 1:**
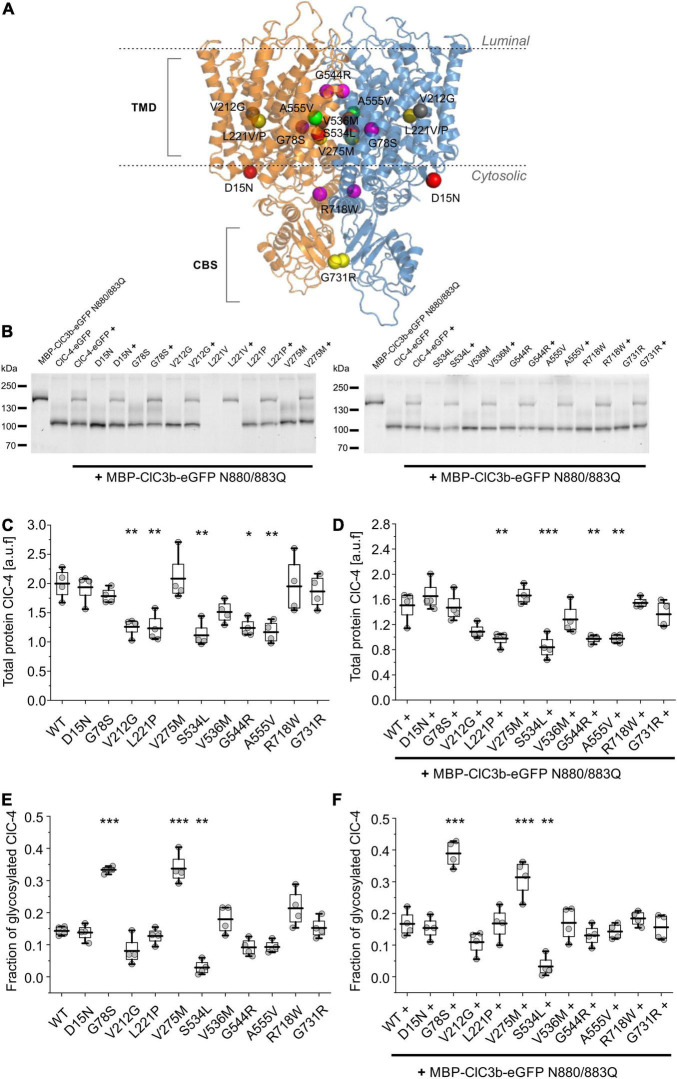
Effects of disease-causing mutations on protein expression and stability. **(A)** Position of amino acid substitutions examined in this study mapped onto the backbone fold of an atomic-resolution model of the human ClC-4 dimer generated using AlphaFold-Multimer (Evans et al., 2021). **(B)** Representative SDS-PAGE analysis of lysates from HEK293T cells expressing WT or mutant ClC-4, alone or with ClC-3b. **(C,D)** Total WT or mutant ClC-4-eGFP protein amounts were estimated by quantifying fluorescence intensities in SDS-PAGE of lysates from HEK293T cells expressing ClC-4 **(C)** alone or **(D)** with ClC-3b (a.u. denotes arbitrary units). **(E,F)** Ratio of the intensities of complex glycosylated to total WT or mutant ClC-4-eGFP fluorescence from HEK293T cells expressing ClC-4 **(E)** alone or **(F)** with ClC-3b. ClC-4 variants were compared with WT using one-way analysis of variance (with Tukey’s HSD *post hoc* testing), ***p < 0.001, **p < 0.01, and *p < 0.05. Data were obtained from four independent experiments and are represented as boxplot boxes indicating upper and lower quartiles and whiskers indicating upper and lower 90%.

We here analyzed a total of 12 *CLCN4* variants that were associated with X-linked intellectual disability and epilepsy in previous studies, G78S, L221V, V536M, G731R (Hu et al., 2016), D15N, V212G, L221P, V275M, S534L, A551V, R718W (Palmer et al., 2018) and G544R (Veeramah et al., 2013). These naturally occurring variants result in amino acid substitutions across the whole protein, from the cytosolic amino-terminus (D15N) to the carboxy-terminal CBS domains (R718W, G731R; Figure 1A). To determine whether disease-causing mutations affect protein expression/stability, we quantified protein expression and glycosylation by SDS-PAGE after the transient expression of eGFP-tagged WT or mutant ClC-4 in mammalian cells (Janssen et al., 2009; Winter et al., 2012; Kovermann et al., 2017). Since ClC-4 can form homodimers, as well as heterodimers with ClC-3 (Guzman et al., 2017), we analyzed cells expressing WT or mutant ClC-4 either alone or together with the lysosomal ClC-3 isoform, ClC-3b (Guzman et al., 2015). Figure 1B shows the migration of WT and mutant ClC-4 under denaturing conditions in the presence or absence of ClC-3b. ClC-4-eGFP migrates as a main band with a molecular weight of approximately 100 kDa. Additional faint higher-molecular-weight bands were sensitive to PNGase F (Supplementary Figure 2), but not to EndoH, indicating that heterologously expressed ClC-4 is complex glycosylated.

There was no band corresponding to full-length L221V ClC-4 protein, but the presence of proteolytic fragments linked to eGFP was consistent with the mutation promoting ClC-4 degradation (Supplementary Figures 1A,B). Heterodimerization with ClC-3b did not prevent proteolysis of the mutant protein L221V (Figure 1B). Figures 1C,D provide eGFP fluorescence levels obtained from such SDS PAGE, which report on expression levels of WT and mutant ClC-4. V212G, L221P, S534L, G544R, and A555V reduce ClC-4 expression levels, whereas the remaining mutations left transporter amounts unaffected (Figure 1C). Relative expression levels of mutant ClC-4 were similar in the presence or absence of co-transfected ClC-3b (Figure 1C vs. Figure 1D). For many membrane proteins, exit from the ER is associated with complex glycosylation. Therefore, we measured glycosylation levels to determine the proportion of WT and mutant ClC-4 exiting the ER (Figures 1E,F). Most disease-associated mutations did not affect complex glycosylation. However, G78S and V275M increased the complex glycosylation of ClC-4 (Figures 1E,F), whereas S534L decreased the level. No differences were observed between cells expressing ClC-4 alone or ClC-4 plus ClC-3b. Therefore, heterodimerization with ClC-3b does not stimulate the exit of ClC-4 from the ER.

### Disease-Causing Mutations Affect the Efficacy and the Voltage Dependence of ClC-4 Cl^–^/H^+^ Exchange

**FIGURE 2:**
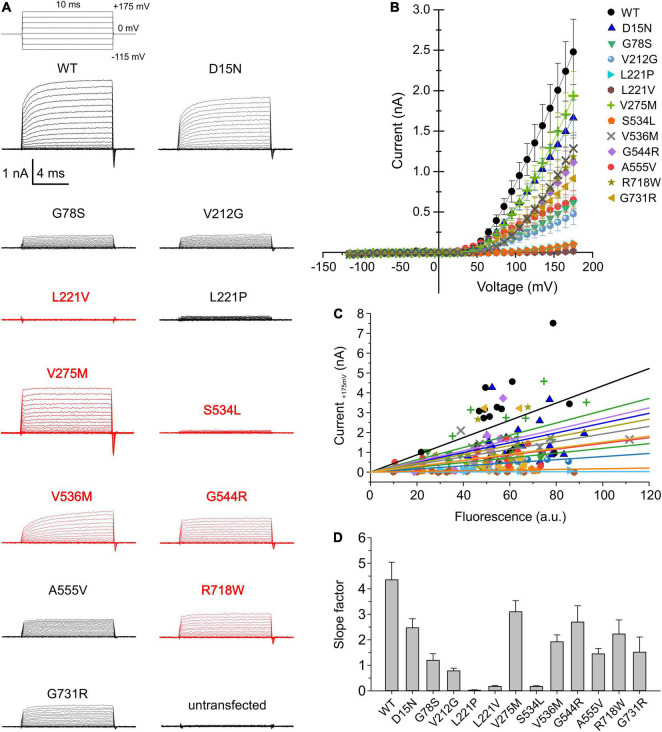
Whole-cell patch clamp analysis of WT and mutant ClC-4 currents. **(A)** Test pulse protocol and representative whole-cell recordings from HEK293T cells expressing WT or mutant ClC-4. **(B)** Steady-state current–voltage relationships for WT and mutant ClC-4 variants, the steady-state current represent the mean value ± s.e.m. **(C)** Current-fluorescence plots for WT and mutant ClC-4. Solid lines represent linear fits (a.u. denotes arbitrary units). **(D)** Slope factors obtained from linear fits to the current-fluorescence plots shown in **(C)**, ClC-4 mutants were compared with WT using one-way analysis of variance (with Tukey’s HSD *post hoc* testing). (WT, *n* = 19; D15N, *n* = 19; G78S, *n* = 10; V212G, *n* = 10; L221P, *n* = 12; L221V, *n* = 12; V275M, *n* = 15; S534L, *n* = 7; V536M, *n* = 12; G544R, *n* = 10; A555V, *n* = 15; R718W, *n* = 12; and G731R, *n* = 11). Data were obtained from four or five independent transfections.

Although ClC-4 is predominantly localized in intracellular compartments of transfected mammalian cells, sufficient transporters are inserted into the plasma membrane to permit the analysis of ClC-4 transport by whole-cell patch-clamp recordings (Friedrich et al., 1999; Alekov and Fahlke, 2009; Guzman et al., 2017). We used whole-cell recordings from transiently transfected HEK293T cells to determine whether disease-associated variants modify ClC-4 transport (Figure 2A; variants associated with epilepsy are shown in red and the others in black). D15N, V275M, V536M, G544R, and R718W reduced current amplitudes only slightly. Currents in cells expressing G78S, V212G, A555V, or G731R ClC-4 resembled WT currents, but with much smaller amplitudes (Figure 2B). L221V, L221P, and S534L reduced ClC-4 currents to the levels measured in untransfected cells (Figures 2A,B). WT and mutant ClC-4 were expressed as eGFP fusion proteins, permitting quantification of transporter expression levels by measuring eGFP fluorescence intensities in individual cells and correlating expression levels and current amplitudes (Schänzler and Fahlke, 2012; Ronstedt et al., 2015; Tan et al., 2017; Kovermann et al., 2022). Figure 2C provides plots of steady-state current amplitudes at +175 mV versus whole-cell fluorescence intensities for HEK293T cells expressing WT or mutant cells. We observed similar expression levels for WT and all mutant ClC-4, however, lower mutant current amplitudes at comparable whole-cell intensities. To obtain averaged and expression-level independent values of the mutation-specific effects on the macroscopic current amplitudes we fitted linear regressions to these plots. The obtained slope factors (Figure 2D) provide macroscopic current amplitudes normalized to expression levels and follow the same order of averaged macroscopic current amplitudes shown in Figure 2B.

**FIGURE 3:**
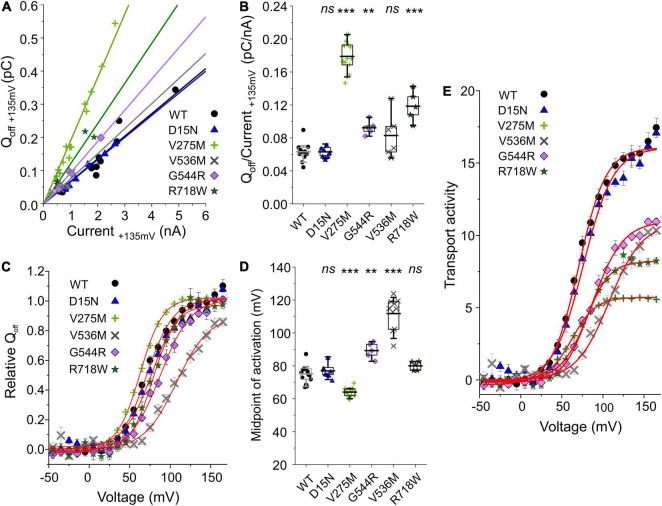
Analysis of WT and mutant ClC-4 capacitive currents. **(A)** Plots of integrated capacitive current amplitudes at 0 mV (*Q*_*off*_) after a prepulse to +135 mV against the transport current amplitude at +135 mV for WT and mutant ClC-4 variants. **(B)** *Q*_*off*_/current ratios for WT and mutant ClC-4 variants (WT, *n* = 14; D15N, *n* = 7; V275M, *n* = 14; V536M, *n* = 6; and G544R, *n* = 5; R718W, *n* = 5). **(C)** ClC-4 activation curves constructed by plotting the mean value ± s.e.m. of the normalized *Q*_*off*_ against the preceding voltage. Solid lines provide fits to single Boltzmann functions. **(D)** Mean values for the midpoint of activation (*V*_0.5_) obtained from Boltzmann fits to the *Q_*off*–_V* relationship for WT and mutant ClC-4 variants (WT, *n* = 14; D15N, *n* = 7; V275M, *n* = 14; V536M, *n* = 10; G544R, *n* = 5; and R718W, *n* = 5). ***p < 0.001, **p < 0.01, and *ns* (not significant). **(E)** Comparison of the transport activities of WT and mutant ClC-4. Transport activities were calculated by dividing normalized Q_*off*_ determined after various prepulses by *Q*_*off*_/current ratios shown in **(B)**. ClC-4 mutants were compared with WT using one-way analysis of variance (with Tukey’s HSD *post hoc* testing). Data were obtained from four or five independent transfections and are presented as means ± s.e.m. and boxplot boxes indicating upper and lower quartiles; whiskers indicate upper and lower 90%.

ClC-4 currents exhibit a characteristic voltage dependence, with currents close to background at negative voltages and pronounced outward rectification and voltage-dependent current activation upon depolarizing voltage steps (Friedrich et al., 1999; Alekov and Fahlke, 2009). Voltage steps from depolarizing pulses back to the holding potential of 0 mV elicit a characteristic capacitive current (Smith and Lippiat, 2010; Grieschat and Alekov, 2012; Guzman et al., 2013; Rohrbough et al., 2018). Capacitive membrane currents originate from the repositioning of charges by membrane proteins within the membrane (Armstrong and Bezanilla, 1974). In many transporters, incomplete transport cycles in the absence of transport substrates generate capacitive currents that disappear after the application of all necessary substrates (Mager et al., 1993; Wadiche et al., 1995). Grieschat and Alekov (2012) demonstrated that a transport-incompetent mutant ClC-5 transporter carrying the E268C mutation can be rescued by site-specific addition of a negative MTSES moiety. E268C ClC-5 transporters generate capacitive currents before modification, and the addition of MTSES converts transport-incompetent transporters that function as capacitors are converted into functional transporters. Capacitive currents by ClC-3, ClC-4, or ClC-5 are therefore often assumed to be generated by transporters that perform incomplete transport cycles (please see Discussing for alternative explanations). Dividing the off-gating capacitive charge (Q_*off*_) by the ionic current amplitude (current) at the preceding voltage (Figures 3A,B) can therefore be used as relative measure of the transport efficiency (Grieschat and Alekov, 2012; Guzman et al., 2013). ClC-4 has significantly lower capacitive currents than ClC-3 and ClC-5 when expressed at the same level and, therefore, is considered the most effective transporter in this CLC branch (Guzman et al., 2013). V275W, G544R, and R718W increased the Q_*off*_/current ratio (Figures 3A,B).

The voltage dependence of transport can be determined by plotting the integrated capacitive currents at 0 mV against the preceding voltages. Fitting Boltzmann functions to these voltage dependences provides a similar half-maximal activation voltage (*V*_0.5_) for WT, D15N, and R718W ClC-4 (WT, +75 ± 2 mV, *n* = 14; D15N, +77 ± 2 mV, *n* = 7; and R718W, +80 ± 1 mV, *n* = 5) (Figures 3C,D). V275M shifts the activation curve by about 11 mV in the hyperpolarizing direction (+64 ± 1 mV, *n* = 14), demonstrating activation at less positive potentials than the WT. In contrast, V536M and G544R cause a change in voltage dependence in the opposite direction (V536M, +112 ± 3 mV, *n* = 10; and G544R, +89 ± 2 mV, *n* = 5) (Figures 3D,E), indicating that V536M and G544R ClC-4 exhibit lower Cl^–^/H^+^ transport rates as WT when operating at the same voltage. To account for the difference in the relative transport to capacitive currents (Figures 3A,B), we divided the capacitive currents measured at 0 mV after different prepulse by the off gating/transport current ratio. This novel parameter – that we will call transport activity hereafter – reports on voltage-dependent changes in transport efficacy. We observed reduced transport activities for V275M, V536M, G544R, and R718W ClC-4 (Figure 3E).

**FIGURE 4:**
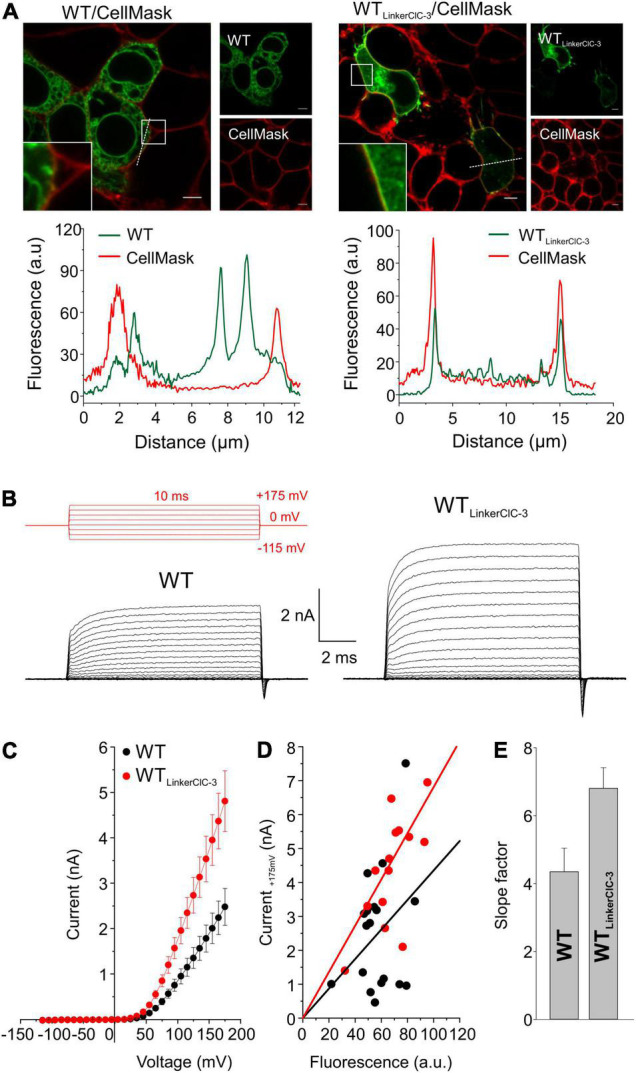
Exchanging the ClC-4 linker region increases ClC-4 surface membrane insertion without affecting ion transport. **(A)** Representative confocal pictures of HEK293T cells co-expressing WT or WT ClC-4_*LinkerClC–*3_ together with the plasma membrane marker CellMask and line scan analyses illustrating that subcellular distribution pattern at both channels (dashed lines). Inset shows areas outlined in the white boxes. Scale bars 5 μm. **(B)** Test pulse protocol (in red) and representative whole-cell recordings from HEK293T cells expressing WT or WT ClC-4_*LinkerClC–*3_. **(C)** Steady-state current–voltage relationships for WT and WT ClC-4_*LinkerClC–*3_, given as means ± s.e.m. **(D)** Current-fluorescence plots for WT and WT ClC-4_*LinkerClC–*3_. Solid lines represent linear fits, (a.u. denotes arbitrary units). **(E)** Slope factors obtained from **(D)**. (WT, *n* = 18 and WT ClC-4_*LinkerClC–*3_, *n* = 15) Data were obtained from four or five independent transfections.

**FIGURE 5:**
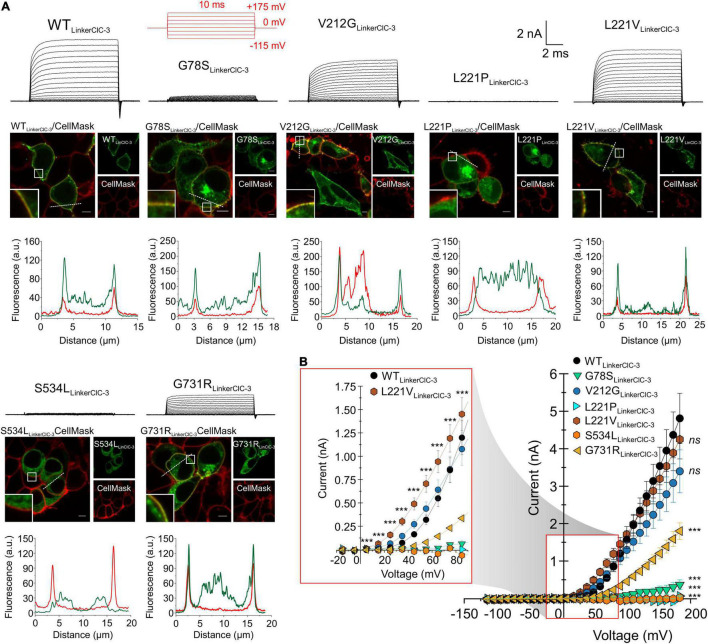
Subcellular expression and transport functions of WT and mutant chimeric ClC-4_*LinkerClC–*3_. **(A)** Test pulse protocol (in red) and representative transport currents (black traces) for chimeric WT, G78S, V212G, L221P, L221V, S534L, or G731R ClC-4_*LinkerClC–*3_ expressed in HEK293T cells (upper panels). Representative confocal images of HEK293T cells co-expressing WT or mutants chimeric ClC-4_*LinkerClC–*3_ together with the plasma membrane marker CellMask (middle panels) and line scan analyses illustrating that subcellular distribution pattern at both channels (dashed lines), (lower panels), (a.u. denotes arbitrary units). Inset shows areas outlined in the white boxes. Scale bars 5 μm. **(B)** Steady-state current-voltage relationships (mean value ± s.e.m.) for WT and mutant ClC-4 chimeric proteins. The inset gives an expanded view of the current-voltage relationship (WT, *n* = 14; G78S, *n* = 10; V212G, *n* = 12; L221P, *n* = 4; L221V, *n* = 12; S534L, *n* = 8; and G731R, *n* = 11). Data were obtained from four or five independent transfections.

Since ClC-4 homodimers are present only at low densities in the plasma membrane, low whole-cell current amplitudes in cells expressing G78S, V212G, L221P, S534L, or G731R ClC-4 may be caused by changes in the subcellular distribution. We increased the surface density of these mutant ClC-4 transporters by exchanging the ClC-4 linker region between the CBS domains with the corresponding ClC-3 sequence (Guzman et al., 2017) to form a chimeric ClC-4_*LinkerClC–*3_ (Figures 4, 5). Figure 4 provides a comparison of the subcellular distribution and the transport function of WT ClC-4 and WT ClC-4_*LinkerClC–*3_. Whereas WT ClC-4-eGFP is mainly localized to the ER, we observed predominant surface membrane insertion after expressing WT ClC-4_*LinkerClC–*3_ (Figure 4A). In agreement with increased surface membrane insertion, the steady-state current (Figures 4B,C) and slope factors in current-fluorescence plots (Figures 4D,E) were about twofold increased by the interlinker substitution. We did not observe any differences in the ratio of capacitive by transport currents (Supplementary Figures 3A,B) or in the voltage dependence of the gating charge movement (Supplementary Figures 3C,D) between WT ClC-4 and WT ClC-4_*LinkerClC–*3_, in agreement with earlier studies (Guzman et al., 2017). These results indicate that exchanging the ClC-4 linker region between the CBS domains with the corresponding ClC-3 sequence leaves transport unaltered, but increases the surface membrane insertion of ClC-4.

**FIGURE 6:**
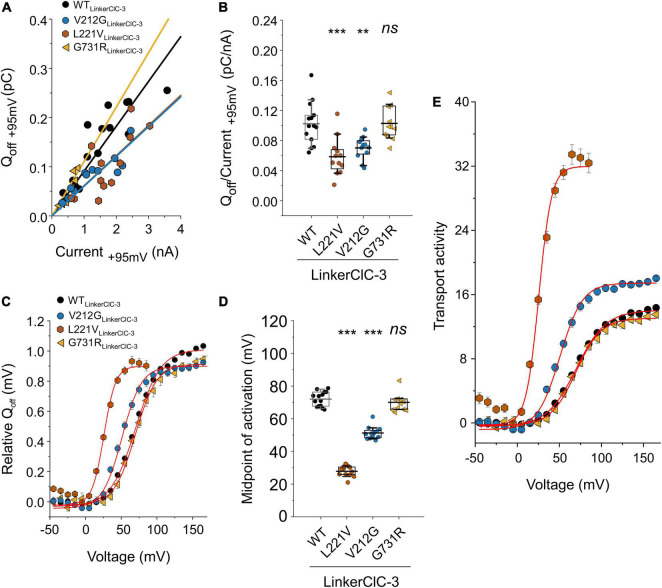
Analysis of WT and mutant chimeric ClC-4_*LinkerClC–*3_ capacitive currents. **(A)** Plots of integrated capacitive current amplitudes at 0 mV (*Q*_*off*_) after a prepulse to +95 mV against transport current amplitudes at +95 mV for WT and mutant chimeric proteins. **(B)** *Q*_*off*_/current ratios for WT and mutant ClC-4 chimeric proteins. **(C)** ClC-4 activation curves constructed by plotting mean value ± s.e.m. of the normalized *Q*_*off*_ against the preceding voltage. Solid lines show fits to single Boltzmann functions. **(D)** Mean values of activation midpoints (*V*_0.5_) obtained from Boltzmann fits to *Q_*off*–_V* relationship for WT and mutant chimeric transporters (WT, *n* = 14; V212G, *n* = 12; L221V, *n* = 12; and G731R, *n* = 11). (WT, *n* = 14; V212G, *n* = 12; L221V, *n* = 12; and G731R, *n* = 11). ***p < 0.001, **p < 0.01, and *ns* (not significant). **(E)** Transport activities of WT and mutant ClC-4 chimeric proteins calculated by dividing normalized *Q*_*off*_ determined after various prepulses by *Q*_*off*_/current ratios shown in **(B)**. Mutant chimeric ClC-4_*LinkerClC–*3_ were compared with ClC-4 WT_*LinkerClC–*3_ using one-way analysis of variance (with Tukey’s HSD *post hoc* testing). Data were obtained from four or five independent transfections and are presented as mean ± s.e.m. and boxplot boxes indicating upper and lower quartiles; whiskers indicate upper and lower 90%.

Exchanging the ClC-4 linker region promoted surface membrane insertion for G78S, V212G, and G731R, but not for L221P and S534L (Figure 5A). It stabilized L221V ClC-4 protein and also promote surface membrane localization for the chimeric L221V (Figure 5A). Moreover, it significantly increased the amplitude of the transport current for V212G, L221V and G731R ClC-4, but not for G78S ClC-4 (Figures 2, 5A,B). V212G and L221V ClC-4_*LinkerClC–*3_ exhibited larger Q_*off*_/late current ratios than WT ClC-4_*LinkerClC–*3_, but unaltered values for G731R ClC-4_*LinkerClC–*3_ (Figures 6A,B). The voltage dependency of the gating charge movement shifted to more negative voltages by about 21 mV for V212G (*V*_0.5_ = + 51 ± 1 mV, *n* = 12) and 44 mV for L221V (*V*_0.5_ = 28 ± 1 mV, *n* = 12) (Figures 5B inset, 6C,D) compared to WT, but was unchanged for G731R. These results suggest a higher transport activity for V212G ClC-4 and possibly also for L221V ClC-4 (Figure 6E); however only under conditions, in which mutant proteins are stabilized by associated proteins. Chimeric transporters carrying G78S, L221P, or S544L and the ClC-3 linker sequence did not produce measurable transport ionic currents (Figures 5A,B).

### Intracellular Trafficking of Mutant ClC-4

**FIGURE 7:**
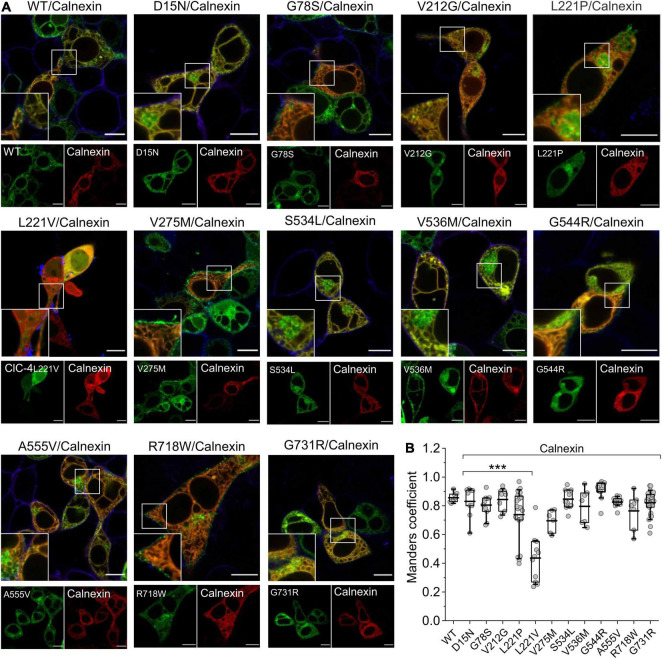
Subcellular localization of WT and mutant ClC-4 homodimers. **(A)** Representative confocal images of HEK293T cells co-expressing WT or mutant ClC-4 together with the endoplasmic reticulum marker calnexin. Note that all mutants localize to the ER; however, the V275M and R718W variants are also present within the plasma membrane, and D15N, V536M, A555V, and S534L also localize to perinuclear structures. Inset shows areas outlined in the white boxes. **(B)** Mean values for Mander’s overlapping coefficients (WT, *n* = 8; D15N, *n* = 9; G78S, *n* = 11; V212G, *n* = 10; L221P, *n* = 20; L221V, *n* = 11; V275M, *n* = 6; S534L, *n* = 11; V536M, *n* = 7; G544R, *n* = 11; A555V, *n* = 11; R718W, *n* = 7; G731R, *n* = 27). ***p < 0.001. ClC-4 mutants plus calnexin were compared with WT ClC-4 plus calnexin using one-way analysis of variance (with Tukey’s HSD *post hoc* testing). Data were obtained from three or four independent transfections and are presented as boxplot boxes indicating upper and lower quartiles; whiskers indicate upper and lower 90%. Scale bar represents 10 μm.

We next studied possible disease-associated alterations in subcellular targeting by confocal imaging of transfected mammalian cells. Homodimeric ClC-4 localizes to the ER, but reaches distinct endosomal compartments in heterodimeric assemblies with various ClC-3 splice variants (Guzman et al., 2017). We first determined the localization of fluorescent ClC-4 fusion proteins in HEK293T cells after co-expression with the ER marker calnexin (Figure 7). Extensive co-localization was apparent between WT ClC-4 and calnexin. All mutants, except for L221V ClC-4, were localized to the ER (Figure 7A). The fluorescence signal for L221V ClC-4 was rather homogenous throughout the cytoplasm, but also present in the nucleus, albeit at a lower intensity. The slight nuclear fluorescence signal may be explained by the formation of soluble fusion proteins of variable size, some of which are small enough to pass through nuclear pores (Hebeisen et al., 2004), consistent with the proteolytic degradation of L221V ClC-4 (Figure 1B). In cells expressing L221V ClC-4_*LinkerClC–*3_-eGFP, a fluorescent signal was apparent in the ER (Supplementary Figure 4), consistent with the expression of the full-length mutant chimeric protein. The subcellular distribution of other mutants was not restricted to the ER: V275M and R718W showed a pronounced plasma membrane localization, and D15N, V536M, A555V, and S534L also localized to structures near to the nucleus (Figure 7).

**FIGURE 8:**
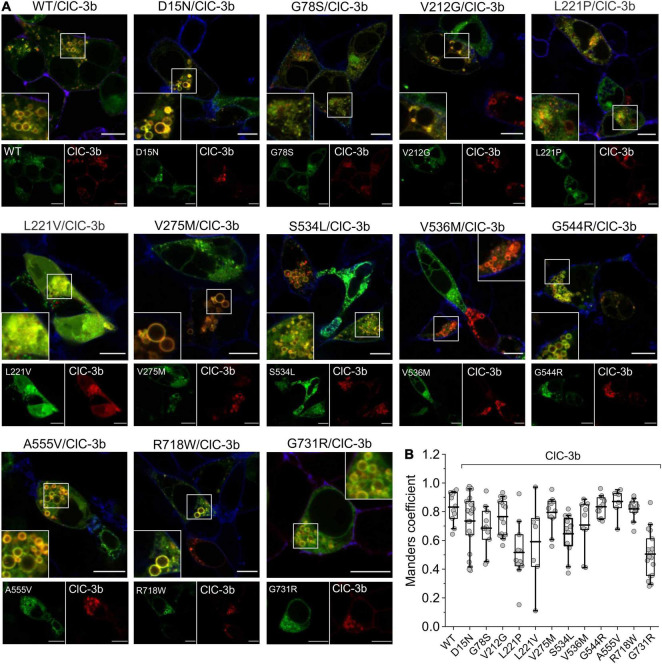
Subcellular localization of ClC-3b/ClC-4 (WT and mutant) heterodimers. **(A)** Representative confocal images of HEK293T cells co-expressing WT or mutant ClC-4 together with ClC-3b. ClC-4 was tagged with eGFP and ClC-3b with mCherry. Inset shows areas outlined in the white boxes. **(B)** Mean values of Mander’s overlapping coefficient analyses. (WT, *n* = 11; D15N, *n* = 21; G78S, *n* = 11; V212G, *n* = 14; L221P, *n* = 11; L221V, *n* = 7; V275M, *n* = 11; S534L, *n* = 12; V536M, *n* = 9; G544R, *n* = 11; A555V, *n* = 7; R718W, *n* = 11; G731R, *n* = 17). ClC-4 mutants plus ClC-3b were compared with WT ClC-4 plus ClC-3b using one-way analysis of variance (with Tukey’s HSD *post hoc* testing). Data were obtained from three or four independent transfections and are presented as boxplot boxes indicating upper and lower quartiles; whiskers indicate upper and lower 90%. Scale bar represents 10 μm.

We next evaluated the subcellular distribution of heterodimers assembled from ClC-3b and mutant ClC-4. Confocal images of HEK293T cells co-expressing WT ClC-4-eGFP and ClC-3b-mCherry showed that the proteins co-localize to enlarged endosomal compartments (Figure 8A; Guzman et al., 2015), with a Mander’s overlap coefficient of 0.83 ± 0.03 (*n* = 11) (Figure 8B). Confocal images of cells co-expressing D15N, V275M, V536M, G544R, or R718W ClC-4 and ClC-3b resembled cells expressing WT ClC-4 and ClC-3b (Figure 8A). Mutant proteins carrying these mutations were detected in close proximity to ClC-3b in endo-lysosomal structures, with similar Mander’s overlap coefficients to those of WT ClC-4 (Figure 8B). In cells expressing L221V ClC-4, we observed fluorescent signals in multiple subcellular compartments, consistent with the presence of eGFP-tagged ClC-4 fragments (Figures 1, 7). The detection of ClC-3b outside lysosomes suggests that some of these ClC-4 fragments may interfere with ClC-3b targeting to the lysosome. Co-expression of ClC-3b and WT ClC-4 is typically associated with enlargement of the lysosomal compartment, which contains the highest density of heterodimers. However, this was not the case for the mutants G78S and L221P or, to a lesser extent, for S534L and G731R (Figure 8A). Co-expression of L221V ClC-4_*LinkerClC–*3_-eGFP and ClC-4-mCherry led to the generation of enlarged lysosomes containing both fluorescent signals (Supplementary Figure 4). Heterodimers of L221P, S534L, or G731R with ClC-3b showed more pronounced residual ER expression compared with WT (Figure 8A).

### Heterodimerization of Mutant ClC-4 With ClC-3

Since confocal microscopy can only provide indirect evidence of homo- or heterodimerization, we used hrCNE to quantify the oligomerization capacity of ClC-4 mutants (Nicke et al., 1999; Wittig et al., 2007; Detro-Dassen et al., 2008; Nothmann et al., 2011). To enable ClC-3b (91 kDa) and ClC-4 (84 kDa) to be distinguished electrophoretically, we increased the size of ClC-3b by attaching a MBP moiety (42.5 kDa) to the amino-terminus (Guzman et al., 2017). Furthermore, as the presence of multiple glycosylation moieties would make it difficult to identify the different native conformations of ClC-3b and ClC-4, we reduced ClC-3b glycosylation by exchanging Asn-880 and Asn-883 for glutamine.

**FIGURE 9:**
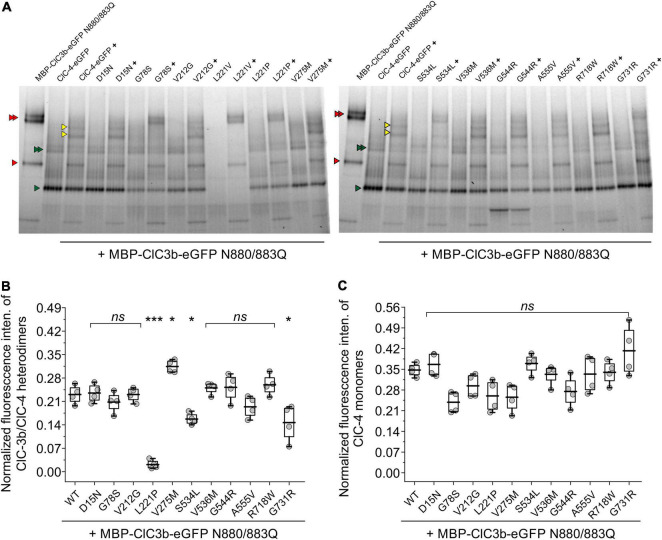
Quantification of the oligomerization capacity of ClC-4 mutant variants. **(A)** hrCNE analysis of ClC-3b or WT/mutant ClC-4 after heterologous expression in HEK293T cells. Red triangles indicate ClC-3b monomer and ClC-3b homodimer bands, green triangles indicate ClC-4 monomer and ClC-4 homodimer bands, yellow triangles indicate heterodimer ClC- 3b/ClC-4 bands. The homodimers, as well as the heterodimer, appear as double bands in the gel due to its glycosylation status. Non-glycosylated protein, lower band and glycosylated protein, upper band. Glycosylated ClC-4 monomer bands are not indicated explicitly but run in between ClC-4 monomer and ClC-3b monomer band. **(B)** Relative fluorescence intensities of ClC-3b/ClC-4 heterodimeric bands. **(C)** Normalized fluorescence intensities of ClC-4 monomers. ***p < 0.001, *p < 0.05, and *ns* (not significant). ClC-4 variants plus ClC-3b were compared with WT plus ClC-3b using one-way analysis of variance (with Tukey’s HSD *post hoc* testing). Data were obtained from four independent transfections/gels and are presented as boxplot boxes indicating upper and lower quartiles; whiskers indicate upper and lower 90%.

Figure 9A shows a representative hrCNE of whole-cell lysates of mammalian cells expressing WT or mutant ClC-4-eGFP with or without MBP-ClC-3b-eGFP N880/883Q. For transfected ClC-3b, two distinct bands of similar intensity were seen by hrCNE (Figure 9A; one representing monomers, lower red triangle, and the other homodimers upper red triangles); in contrast, transfected ClC-4 was predominantly monomeric, lower green triangle, with only a faint band representing homodimers, upper green triangles (Figure 9A). When the proteins were co-expressed, heterodimerization resulted in two additional bands (yellow triangles in Figure 9A). We used the ratio of eGFP intensity for these particular bands to the sum of intensities for all fluorescent bands to quantify the heterodimerization capability of ClC-4. The capabilities of ClC-4 D15N, G78S, V212G, V275M, V536M, G544R, A555V, and R718W to heterodimerize with ClC-3b were similar to the WT. In contrast, L221P, S534L, and G731R ClC-4 had lower capabilities. If we assume that ClC-4 requires ClC-3 for export from the ER to endo-lysosomal compartments (Guzman et al., 2017), our findings suggest that L221P, S534L, and G731R ClC-4 are less likely to be inserted into endo-lysosomal membranes. V275M ClC-4 had an increased probability of heterodimerization with ClC-3b and, thus, this mutation may increase the number of ClC-4 transporters in the endo-lysosome. Since L221V impaired ClC-4 stability, we excluded this mutation from the analysis.

## Discussion

X-linked intellectual disability and epilepsy represents a group of syndromes with large variability in the severity of symptoms (Ropers and Hamel, 2005). The discovery of causative *CLCN4* mutations in several patients with distinct symptom severity (Table 1) provides the opportunity to link changes in endo-lysosomal Cl^–^/H^+^ exchange with specific neurological symptoms (Hu et al., 2016; Palmer et al., 2018). Here we examined the functional consequences of 12 disease-associated *CLCN4* mutations that result in the substitution of single amino acids (Hu et al., 2016; Palmer et al., 2018) using biochemical, microscopy, and electrophysiological approaches. Our analysis permitted classification of the tested disease-associated ClC-4 mutations into three groups: one group encompassing mutations (D15N, V275M, V536M, G544R, A555V, and R718W) with only subtle changes in transport properties and almost normal subcellular localization, a second group causing pronounced reductions in endosomal ClC-4 transport (G78S, L221P, L221V, and S534L), and a third group (V212G and G731R) mainly affecting transporter biogenesis or trafficking.

**TABLE 1:** Mutation-associated changes in ClC-4 function, trafficking, oligomerization, and clinical symptoms in patients carrying these mutations.

Variant	Protein dysfunction					Functional category	Clinical phenotype in humans (Palmer et al., 2018)
	Trafficking	Dimerization with ClC-3	*V*_0.5_	Transport efficiency	*Iss* at + 175 mV		
p.D15N	Increased ER staining Perinuclear staining	Not altered	77.0 ± 1.6 mV	Not altered	Not altered	Normal function, normal homodimer localization,	Borderline intellectual disability, delayed speech, infantile hypotonia, congenital diaphragmatic hernia, bilateral hip dysplasia, umbilical hernia, and infantile failure to thrive
p.G78S	Not altered	Not altered	ND	ND	Reduced	Loss-of-function, normal trafficking	Moderate intellectual disability and anxiety
p.V212G	Increased ER staining Perinuclear staining	Not altered	51.2 ± 1.1 mV	Increased	Reduced	Gain-of-transport function, altered trafficking	Mild to moderate intellectual disability, delayed speech, self-abusive and obsessive, compulsive behavior, anxiety, and depression.
p.L221P	Increased ER staining Perinuclear staining	Reduced	ND	ND	Reduced	Heterodimerization	Moderate intellectual disability, delayed speech, self-abusive behavior, sleep initiation disorder, infantile hypotonia, and unsteady wide-base gait
p.L221V	Impaired	Reduced	27.8 ± 0.9 mV	Increased	Not altered	Loss-of-transport function, altered trafficking as homo- and heterodimer, impaired heterodimerization	Mild to moderate intellectual disability, delayed speech, depression, bipolar and seizures disorder, epilepsy, neurological features, and gastroesophageal reflux
p.V275M	Increased ER staining Perinuclear staining	Not altered	64.0 ± 1.0 mV	Reduced	Not altered	Normal trafficking, moderate impairment of Cl^–^-H^+^ transport	Moderate to severe intellectual disability, delayed speech, epilepsy, brisk patella reflexes
p.S534L	Increased ER staining Perinuclear staining	Slightly reduced	ND	ND	Reduced	Loss-of-function or altered trafficking	Severe intellectual disability, delayed speech, epilepsy, infantile hypotonia, cortical visual impartment and upper limb hypertonia and spasticity at the age of 3 years
p.V536M	Increased ER staining Perinuclear staining	Not altered	112.0 ± 3.1 mV	Not altered	Reduced	Altered homodimer trafficking, normal heterodimerization, moderately impaired transport	Moderate to severe intellectual disability delayed speech, aggressive behavior, hyperactivity, epilepsy, progressive spasticity, and progressive unsteady gait
p.G544R	Not altered	Not altered	89.2 ± 2.1 mV	Reduced	Reduced	Heterodimerization	Severe intellectual disability, epilepsy, infantile hypotonia, and dystonic posturing
p.A555V	Increased ER staining Perinuclear staining	Not altered	ND	ND	Reduced	Loss-of-transport function normal homodimer trafficking	Mild to moderate intellectual disability, delayed speech, exotropia, feeding difficulties, and constipation
p.R718W	Increased ER staining Perinuclear and surface membrane staining	Not altered	80.0 ± 1.2 mV	Reduced	Reduced	Moderately impaired transport function, normal trafficking	Severe intellectual disability, delayed speech, drooling and self-abusive behavior, epilepsy, infantile hypotonia, feeding difficulties in infancy and scoliosis
p.G731R	Increased ER staining Perinuclear staining	Impaired	70.0 ± 1.6 mV	Not altered	Reduced	Normal transport, change in homodimer trafficking, normal heterodimer trafficking	Severe to profound intellectual disability, delayed speech, hyperactivity, slowness and apathy, perseveration and anxiety, infantile hypotonia, and strabismus

*PM, plasma membrane; PS, perinuclear structures; ND, = non-determined; Iss, steady-state anion current; **V_0_._5_** ClC-4 WT = 75.0 ± 1.05 mV or ClC-4_linkerClC–3_ = 72.0 ± 1.2 mV (Palmer et al., 2018).*

We used SDS-PAGE and confocal imaging to describe protein expression, stability, and subcellular distribution of WT and mutant ClC-4. While D15N, G78S, V275M, V536M, R718W, and G731R ClC-4 were indistinguishable from WT in these analyses, we observed reduced full-length expression levels of V212G, L221P, S534L, G544R, and A555V ClC-4. A small fraction of WT ClC-4 was complex glycosylated. G78S and V275M mutations led to increased and the S524L mutation to reduced complex glycosylation, indicating changes in intracellular trafficking of the mutant proteins. Furthermore, confocal imaging revealed increased insertion of V275M ClC-4 into the plasma membrane (Figure 7). L221V was the only tested mutation with pronounced effects on expression and stability of ClC-4. In lysates from cells transfected with an L221V ClC-4 expression plasmid only protein fragments were detected, indicating decreased protein stability (Figure 1). Confocal imaging of cells expressing L221V ClC-4 with or without ClC-3b revealed fluorescence signals in multiple cell compartments, including the nucleus, consistent with the proteolytic generation of diffusible ClC-4 fragments (Figures 7, 8). These results suggest that L221V mutation leads to complete loss of function of ClC-4; however, exchange of the carboxy-terminal inter-CBS linker stabilized and rescued the function of the mutant (Figures 5, 6). Therefore, we cannot exclude the possibility that endogenous L221V ClC-4 function is retained in affected patients owing to its association with undefined chaperones. In agreement with such stabilization of L221V ClC-4, expression in oocytes resulted in measurable currents (Hu et al., 2016).

In whole-cell patch-clamp experiments, currents were only slightly reduced for five mutants, D15N, V275M, V536M, G544R, and R718W, permitting detailed analysis of disease-associated changes in ClC-4 ion transport (Figures 2, 3). A distinctive feature of ClC-3, ClC-4, and ClC-5 is strong outward rectification with virtually no inward currents at negative potentials; however, none of the tested mutations affected rectification. CLC Cl^–^/H^+^ exchangers generate voltage and time-dependent currents in response to voltage steps. This behavior might be due to processes that switch transporters from an inactive into an active form, resembling opening and closing of ion channels (Alekov and Fahlke, 2009; De Stefano et al., 2013; Pusch and Zifarelli, 2021). Alternatively, there might be voltage-dependent steps in the transport cycle that become rate-limiting under certain conditions and determine the time course of transport currents. Both mechanisms remain hypothetical, and neither of these possible explanations has been excluded so far. None of the studied mutations causes major alterations in the voltage dependence or kinetics of transport or capacitive currents.

All CLC-type Cl^–^/H^+^ exchangers generate capacitive currents (Smith and Lippiat, 2010; Grieschat and Alekov, 2012; Guzman et al., 2013; Rohrbough et al., 2018; Pusch and Zifarelli, 2021), whose mechanistic basis has remained unclear (Zifarelli et al., 2012; Pusch and Zifarelli, 2021). Capacitive currents generated by incomplete transport cycles have been reported for many secondary active transporters (Loo et al., 1993; Mager et al., 1993; Wadiche et al., 1995; Kovermann et al., 2017). For CLC transporters, two conserved glutamates, called gating and transport glutamate, are crucially involved in proton transport. Neutralizing the transport glutamate abolishes ClC-3, ClC-4, and ClC-5 transport (Smith and Lippiat, 2010; Guzman et al., 2013) and results in prominent capacitive currents by such mutants. Site-specific MTS modification of E268 ClC-5, in which the transport glutamate was substituted by cysteine, converts the capacitor in a transporter (Grieschat and Alekov, 2012), providing strong support for incomplete transport cycles as the basis of capacitive currents. These results support the notion that capacitive currents in CLC transporters are generated by transporters that perform only incomplete transporters, and that ratios of transport to capacitive currents can be used as an estimate of transport efficacy (Guzman et al., 2013). We used this analysis to estimate transport efficacy for WT and mutant ClC-4 and found reduced transport efficacy at +135 mV by three of the 12 mutations, V275M, R718W, and G534R.

The alternative explanation for the capacitive currents, i.e., that capacitive currents represent gating currents associated with transport activation, predicts a similar change in mutant transport rates. Since CLC capacitive currents are linked to transitions in the transport cycle (Zifarelli et al., 2012; Pusch and Zifarelli, 2021) and since none of these mutations caused major alterations of the kinetics of the capacitive currents, the capacitive charge can be used to provide an estimate of the number of transporters. In this mechanistic framework, increased charge by current ratios also corresponds to reduced transport efficacies.

Cl^–^/H^+^ exchange by ClC-4 is voltage-dependent (Alekov and Fahlke, 2009), and the voltage dependence of ion transport can be assessed by plotting capacitive currents against the preceding voltages (Figures 3C,D; Smith and Lippiat, 2010; Grieschat and Alekov, 2012). Whereas the V275M mutation shifted voltage dependence to less positive potentials, V536M and G544R caused an opposite shift to more depolarized potentials. Moreover, hrCNE analysis showed that V275M increased the percentage of heterodimers formed with ClC-3b (Figure 9): the remaining mutations of this group have no obvious effects on the subcellular localization (Figure 7) nor the capability to interact with ClC-3 (Figures 8, 9). D15N substitution did not affect the functional characteristics of ClC-4 in our assays; however, whole-cell currents were reduced with unaltered protein expression (Figures 1, 2). We conclude that D15N, V275M, V536M, G544R, and R718W moderately reduce ClC-4-mediated Cl^–^/H^+^ transport at the ER or at other parts of the endo-lysosomal system under physiological conditions.

Most ClC-4 homodimers remain in the ER, with only a small proportion exported from the ER and inserted into the plasma membrane. Since reduced macroscopic current amplitudes of some mutant proteins might be due to changes in ER export, we removed a previously identified ER retention signal in the inter-CBS domain (Guzman et al., 2017) in mutants associated with complete loss-of-function, when inserted into the WT ClC-4 sequence (G78S, V212G, L221V, S534L, and G731R; Figure 3). Exchanging the ClC-4 linker region between the CBS domains with the corresponding ClC-3 sequence increases current amplitudes of chimeric V212G, L221V, and G731R ClC-4, but leaves currents by chimeric G78S, L221P, and S534L ClC-4 unaffected (Figure 5). Cells expressing V212G and L221V ClC-4 have large current amplitudes with slightly reduced relative capacitive currents that are activated at lower positive voltages, similar to WT (Figures 5A,B). Thus, both V212G and L221V variants have enhanced ClC-4 transport at physiological voltages (Figures 5, 6). However, L221V, as well as V212G, reduce expression levels (Figure 1). We, therefore, conclude that none of the mutations results in gain-of-function mutation of ClC-4.

Co-expression of WT ClC-4 with the lysosomal ClC-3 splice variant ClC-3b (Guzman et al., 2015) result in enlarged lysosome (Figure 8). Currents by chimeric G78S and L221P ClC-4_*LinkerClC–*3_ are very small (Figure 5), and G78S and L221P prevent enlargement of lysosome in cells expressing mutant ClC-4 with ClC-3 (Figure 8). The L221P and S534L mutations reduced the ability of ClC-4 to heterodimerize with ClC-3 in biochemical assays (Figure 9); despite this, co-expression of S534L ClC-4 and ClC-3b still led to enlarged vesicles. L221P, S534L, and A555V, but not G78S, reduce expression levels of ClC-4 (Figures 1C,D). Increased complex glycosylation of G78S ClC-4 indicated additional trafficking defects (Figure 1). The S534L mutation reduced the level of complex glycosylation (Figure 1), consistent with altered trafficking. Cells expressing A555V ClC-4 exhibited only very small transport currents but developed enlarged lysosomes upon ClC-3b co-expression (Figures 2, 8). However, confocal imaging of A555V ClC-4 showed ER localization with additional perinuclear fluorescence signals (Figure 7), which might explain the reduced transport currents. We conclude that G78S, L221P, S534L, and A555V reduce ClC-4 Cl^–^/H^+^ exchange via a number of mechanisms, such as impaired expression, impaired maturation, altered heterodimerization or function.

Patients carrying either of the two mutations that significantly decrease endosomal Cl^–^/H^+^ transport, G78S, and L221P, have only moderate intellectual disability without epilepsy (Table 1). This finding is consistent with the moderate disease symptoms observed in patients carrying *CLCN4* nonsense mutations (Palmer et al., 2018). Patients carrying the L221V mutation suffer from epilepsy and mild-moderate intellectual disability; thus, their phenotype more closely resembles those of patients with mutations that reduce Cl^–^/H^+^ exchange than those with *CLCN4* loss-of-function mutations. S534L and A555V, which also cause pronounced decreases in macroscopic currents in HEK293T cells are both associated with moderate to severe intellectual disability and epilepsy. Of the mutations causing only minor effects on transport (V212G, V275M, V536M, G544R, G731R, and R718W), there was no correlation between the severity of functional changes and the neurological phenotype of affected patients. Mutations with only slight effects on ClC-4 transport are found in patients with (V275M, V536M, G544R, and R718W) or without (D15N, V212G, and G731R) epilepsy. The D15N mutation, which had little effect on ClC-4 function in our analyses, causes only very mild symptoms in patients. Patients carrying the G544R, R718W, or the G731R mutations had severely impaired intellectual performances, but the mutations caused only minor changes in Cl^–^/H^+^ exchange in our functional assays. The V275M mutation increases the ability of ClC-4 to form heterodimers with ClC-3b and is, thus, expected to increase endo-lysosomal ClC-4 Cl^–^/H^+^ transport levels; this mutation was identified in a patent with epilepsy and moderate-severe intellectual disability.

In summary, we performed a detailed functional analysis of the effects of 12 *CLCN4* mutations identified in patients with X-linked intellectual disability and epilepsy on ClC-4 transport, subcellular localization, and heterodimerization with ClC-3. Surprisingly, there are mutations associated with severe intellectual impairments and epilepsy that cause only minor biophysical changes to ClC-4 and mutations causing less severe symptoms, but pronounced alteration in biophysical properties. ClC-4 lacks endosomal targeting signals (Stauber and Jentsch, 2010) and requires association with ClC-3 for insertion into endosomal membranes (Guzman et al., 2017). However, only three of the mutations (L221P, V275M, and G731R) affected heterodimerization with ClC-3. Our results illustrate a lack of correlation between alterations that can be studied in heterologous expression systems and complex neurological phenotypes in these genetic syndromes. It appears likely that impaired ClC-4 functions are either compensated or aggravated by additional, as-yet unidentified partner proteins, compensatory processes, and other genetic factors. Alternatively, disease-associated *CLCN4* mutations might modify processes during development that require tightly regulated Cl^–^/H^+^ exchange and are, therefore, affected by even small changes in ClC-4 transport rates or membrane densities. More complex experimental systems, such as cell or animal models, are required to address these possibilities. However, despite its limitations, our description of multiple changes in CLC-mediated Cl^–^/H^+^ exchange by disease-associated mutations represents a first step toward understanding the molecular basis of X-linked intellectual disability and epilepsy.

## Data Availability Statement

The original contributions presented in the study are included in the article/Supplementary Material, further inquiries can be directed to the corresponding author/s.

## Ethics Statement

All experiments were performed according to the German regulation for genetically modified organisms of Risk Group 1 (§§6.7 GenTG; GenTSV, Appendix 2), Aktenzeichen 53.02.01-K-1.119/15.

## Author Contributions

RG and CF conceived the project. RG and CF wrote the manuscript, with input from all co-authors. RG, JS-M, SB-P, and AF performed the research and analyzed the data. All authors contributed to the article and approved the submitted version.

## Conflict of Interest

The authors declare that the research was conducted in the absence of any commercial or financial relationships that could be construed as a potential conflict of interest. The handling editor TS declared a past co-authorship with several of the authors RG, JS-M, and CF.

## Publisher’s Note

All claims expressed in this article are solely those of the authors and do not necessarily represent those of their affiliated organizations, or those of the publisher, the editors and the reviewers. Any product that may be evaluated in this article, or claim that may be made by its manufacturer, is not guaranteed or endorsed by the publisher.

## Footnotes

1
http://n2t.net/addgene:55005

2
https://github.com/deepmind/alphafold
