# Supplementary material for: Functional Characterization of CLCN4 Variants Associated With X-Linked Intellectual Disability and Epilepsy
Source: Front Mol Neurosci. 2022 May 31;15:872407. doi: 10.3389/fnmol.2022.872407 (PMC9198718; doi:10.3389/fnmol.2022.872407)
Supplement: Supplementary file 1 [file Image_1.pdf]

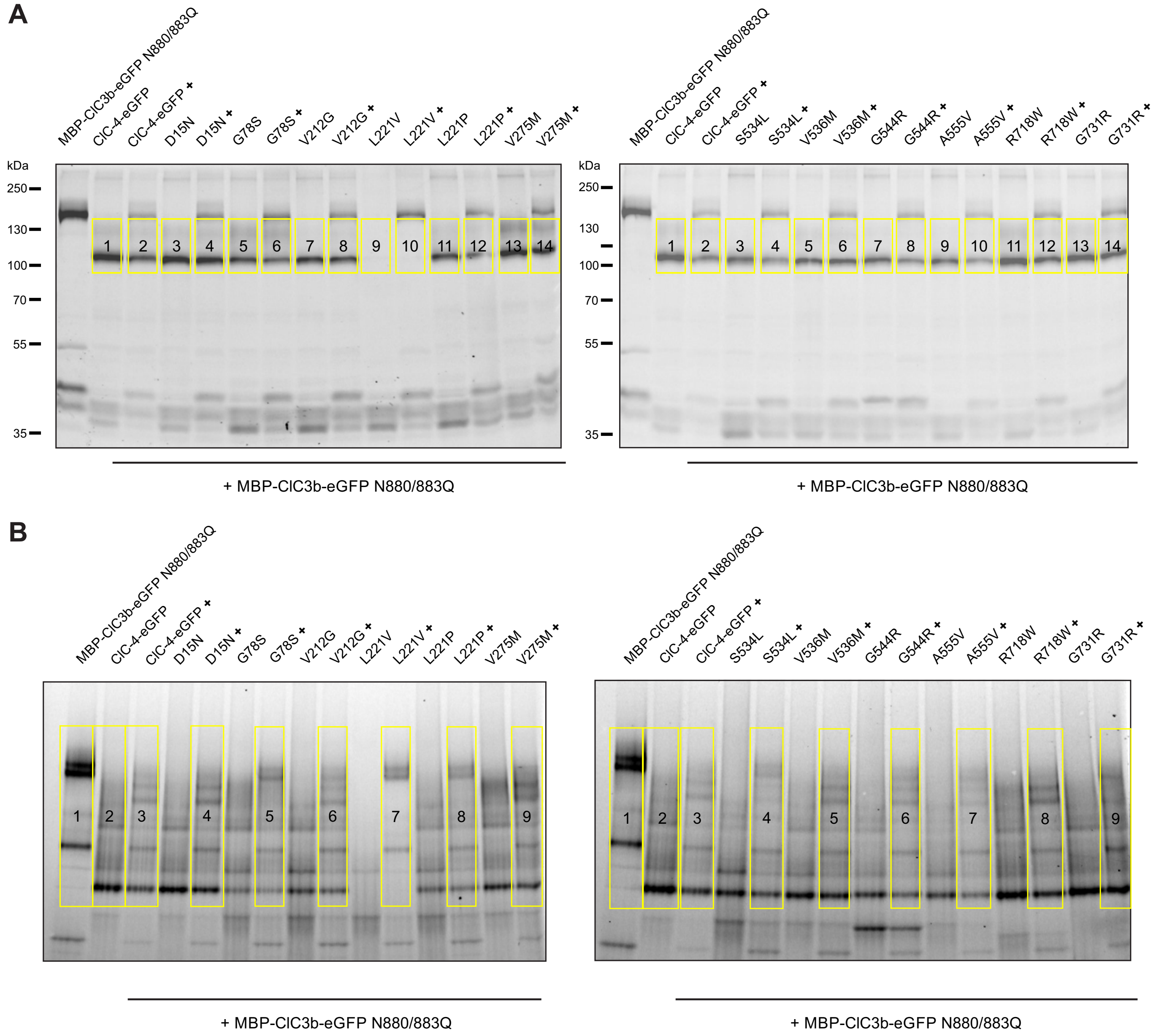

Supplementary Fig. 1

Supplementary Figure 1. Representative SDS and hrCNE gels used for protein quantification. Gel images obtained with a fluorescence gel scanner **(A)** SDS gels and **(B)** hrCNE gels were opened with ImageJ 1.44 software and displayed in black and white. Using the Brightness and Contrast tool, the appearance of the entire gel was adjusted. ROIs are indicated as boxes. See Material and Methods for analysis procedure.
