# Supplementary material for: Functional Characterization of CLCN4 Variants Associated With X-Linked Intellectual Disability and Epilepsy
Source: Front Mol Neurosci. 2022 May 31;15:872407. doi: 10.3389/fnmol.2022.872407 (PMC9198718; doi:10.3389/fnmol.2022.872407)
Supplement: Supplementary file 2 [file Image_2.pdf]

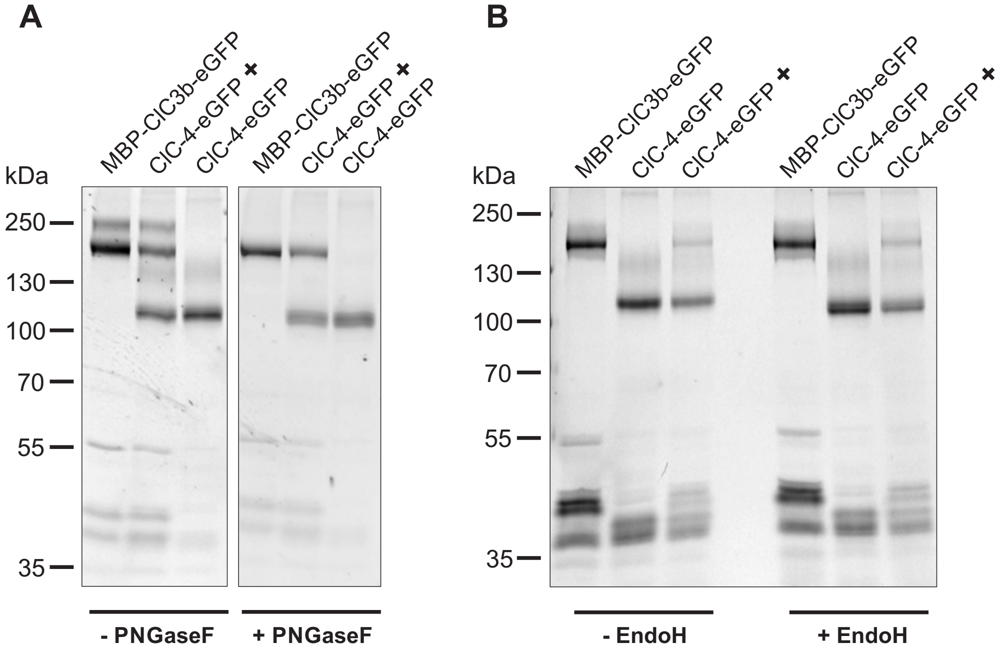

Supplementary Fig. 2

Supplementary Figure 2. Complex glycosylation of ClC-3b and ClC-4. Representative SDS-PAGE of lysates from HEK292T cells expressing WT ClC-3b or ClC-4 alone or together. Higher-molecular-weight ClC-3b and ClC-4 protein bands were removed by incubation of whole-cell lysates with PNGase F **(A)**, but not with EndoH **(B)**, indicating that they represent complex glycosylated ClC-4.
