# Supplementary material for: Functional Characterization of CLCN4 Variants Associated With X-Linked Intellectual Disability and Epilepsy
Source: Front Mol Neurosci. 2022 May 31;15:872407. doi: 10.3389/fnmol.2022.872407 (PMC9198718; doi:10.3389/fnmol.2022.872407)
Supplement: Supplementary file 3 [file Image_3.pdf]

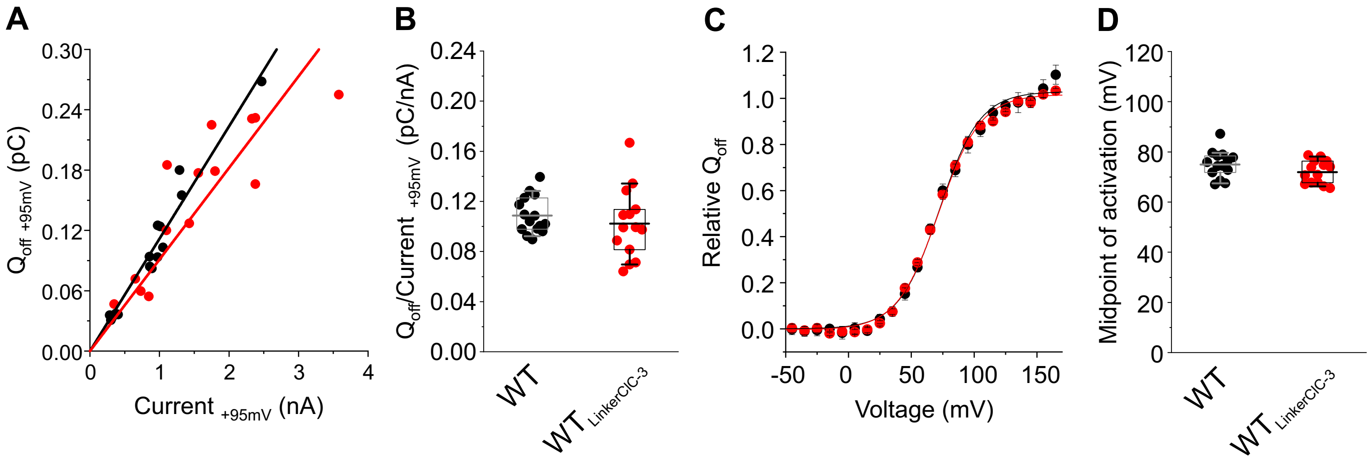

Supplementary Fig. 3

Supplementary Figure 3. ClC-4 and ClC-4$_{LinkerClC-3}$ do not differ in transport properties. **(A)** Plot of integrated capacitive current amplitudes at 0 mV (Q_{off}) after a prepulse to +95 mV against transport current amplitudes at +95 mV for ClC-4 and ClC-4$_{LinkerClC-3}$. **(B)** Q_{off}/current ratios for ClC-4 and ClC-4$_{LinkerClC-3}$. **(C)** ClC-4 and ClC-4$_{LinkerClC-3}$ activation curves constructed by plotting mean value ± s.e.m. of the normalized Q_{off} against the preceding voltage. Solid lines show fits to single Boltzmann functions. **(E)** Mean values of activation midpoints ($V_{0.5}$) obtained from Boltzmann fits to Q_{off}–V relationship for WT and WT ClC-4$_{LinkerClC-3}$. (WT, n = 15 and WT ClC-4$_{LinkerClC-3}$, n = 15) Data were obtained from four or five independent transfections.
