# Supplementary material for: Functional Characterization of CLCN4 Variants Associated With X-Linked Intellectual Disability and Epilepsy
Source: Front Mol Neurosci. 2022 May 31;15:872407. doi: 10.3389/fnmol.2022.872407 (PMC9198718; doi:10.3389/fnmol.2022.872407)
Supplement: Supplementary file 4 [file Image_4.pdf]

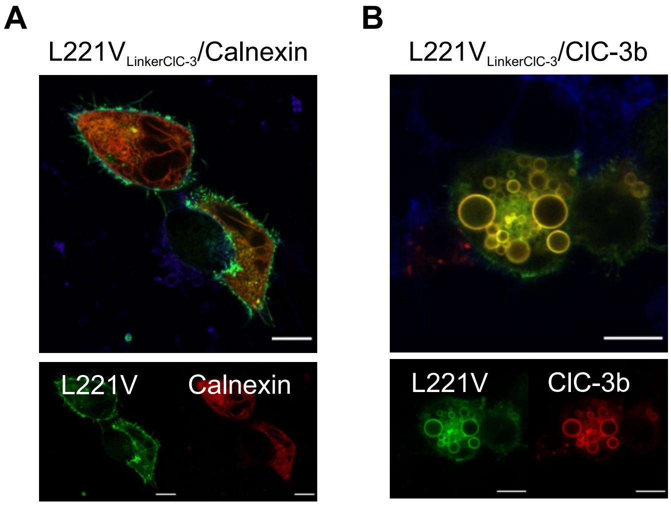

Supplementary Fig. 4

Supplementary Figure 4. Subcellular distribution of the L221V ClC-4 chimeric protein. (A,B) Confocal images of HEK293T cells expressing the chimeric mutant $L221V_{LinkerClC-3}$ alone show extensive plasma membrane localization **(A)** and **(B)** overlapping with ClC-3b in co-transfected cells. Confocal experiments were performed in triplicate. Scale bar represents 10 µm.
